# Association of Adjuvant Chemotherapy With Overall Survival Among Women With Small, Node-Negative, Triple-Negative Breast Cancer

**DOI:** 10.1001/jamanetworkopen.2020.16247

**Published:** 2020-09-14

**Authors:** Oluwadamilola T. Oladeru, Anurag K. Singh, Sung Jun Ma

**Affiliations:** 1 Department of Radiation Oncology, Massachusetts General Hospital, Boston; 2 Department of Radiation Medicine, Roswell Park Comprehensive Cancer Center, Buffalo, New York

## Abstract

This cohort study uses data from the National Cancer Database to evaluate overall survival rates of women who were diagnosed between 2010 and 2015 with small, node-negative, triple-negative breast cancer and were treated with or without adjuvant chemotherapy.

## Introduction

For patients with small (pT1) and node-negative, triple-negative breast cancer (TNBC), the role of chemotherapy has not been prospectively evaluated.^1^ Currently there is a dichotomy between aggressive adjuvant treatment based on tumor biology and deescalation based on tumor size.^1,2^ The National Comprehensive Cancer Network guideline does not recommend adjuvant therapy for pT1aN0 TNBC, although it may be considered in select patients with high-risk features.^1^ Similarly, adjuvant chemotherapy for pT1bN0 TNBC is given on the basis of clinical discretion.^1^ Because of the gap in knowledge, length of survivorship, and the need for consensus guidelines on this subtype, we sought to investigate the overall survival benefit of adjuvant chemotherapy in patients with small, node-negative TNBC.

## Methods

This cohort study was approved by the Roswell Park Comprehensive Cancer Center’s institutional review board. The National Cancer Database was queried for female patients with pT1aN0 or pT1bN0 TNBC who were diagnosed between 2010 and 2015 and treated with or without adjuvant chemotherapy. Informed consent was waived because the data are deidentified and publicly available via an online application process. This study followed the Strengthening the Reporting of Observational Studies in Epidemiology (STROBE) reporting guideline.

*P* values were 2-sided and calculated using the Fisher exact test and Mann-Whitney *U* test. Statistical significance was set at *P* < .05. Overall survival (OS) was evaluated using the Kaplan-Meier method and Cox multivariable analysis. Heterogeneity in treatment effects was assessed using interaction term analysis and subgroup analysis. Propensity score matching was based on the nearest neighbor method in a 1:1 ratio without a replacement. The standardized difference of variables was less than 0.1, suggesting appropriate matching.^3^ Sensitivity analysis was also performed for further evaluation of chemotherapy. Statistical analysis was performed using R statistical software version 3.6.1 (R Project for Statistical Computing) from March to May 2020. See the eAppendix in the Supplement for additional methods.

## Results

A total of 16 180 patients with a median (interquartile range) age of 61 (52-69) years met our inclusion criteria, including 9498 and 6682 patients with and without chemotherapy, respectively. The median (interquartile range) follow-up was 41.6 (24.3-62.0) months. On Cox multivariable analysis, chemotherapy was not associated with improved OS (hazard ratio [HR], 0.99; 95% CI, 0.87-1.13; *P* = .92). Interaction analysis showed the interaction of chemotherapy with tumor size (interaction *P* < .001) and age (interaction *P* < .001). On subgroup analysis to evaluate effect sizes, chemotherapy was associated with worse mortality among patients with pT1a tumors (HR, 1.46; 95% CI, 1.17-1.82; *P* < .001) and among younger patients compared with older patients (age <50 years: HR, 1.77; 95% CI, 1.04-3.00; *P* = .04; age 50-75 years: HR, 0.91; 95% CI, 0.78-1.05; *P* = .21; and age >75 years: HR, 0.83; 95% CI, 0.59-1.16; *P* = .28) but associated with improved OS among patients with pT1b tumors (HR, 0.74; 95% CI, 0.63-0.87; *P* < .001). Similar findings were noted in matched pairs of 1674 patients with pT1a tumors (HR, 1.43; 95% CI, 1.07-1.92; *P* = .02), 1969 patients with pT1b tumors (HR, 0.61; 95% CI, 0.48-0.78; *P* < .001), 432 patients younger than 50 years (HR, 2.16; 95% CI, 1.17-3.99; *P* = .01), and 2851 patients aged 50 years and older (HR, 0.82; 95% CI, 0.67-1.00; *P* = .05) (Table and Figure). Sensitivity analyses using Cox multivariable analysis showed worse mortality with chemotherapy for patients younger than 50 years who had pT1a tumors (HR, 3.11; 95% CI, 1.55-6.22; *P* = .001), while improved OS was seen with chemotherapy for patients aged 50 years or older who had pT1b tumors (HR, 0.72; 95% CI, 0.61-0.85; *P* < .001).

**Table.** Baseline Characteristics for Matched Cohorts^a^

Characteristic	Patients, No. (%)								
	pT1a (n = 1674)		pT1b (n = 1969)		Age <50 y (n = 432)			Age ≥50 y (n = 2851)	
	No chemotherapy	Chemotherapy	No chemotherapy	Chemotherapy	No chemotherapy	Chemotherapy	No chemotherapy		Chemotherapy
Facility volume									
Low	119 (7.1)	130 (7.8)	143 (7.3)	137 (7.0)	31 (7.2)	45 (10.4)	183 (6.4)		183 (6.4)
Intermediate	336 (20.1)	341 (20.4)	480 (24.4)	489 (24.8)	95 (22.0)	109 (25.2)	629 (22.1)		635 (22.3)
High	1219 (72.8)	1203 (71.9)	1346 (68.4)	1343 (68.2)	306 (70.8)	278 (64.4)	2039 (71.5)		2033 (71.3)
Facility type									
Nonacademic	1040 (62.1)	1053 (62.9)	1382 (70.2	1397 (70.9)	223 (51.6)	207 (47.9)	1954 (68.5)		1958 (68.7)
Academic	583 (34.8	558 (33.3)	564 (28.6)	548 (27.8)	123 (28.5)	121 (28.0)	897 (31.5)		893 (31.3)
Not available	51 (3.0)	63 (3.8)	23 (1.2)	24 (1.2)	86 (19.9)	104 (24.1)	0		0
Age, y									
<50	274 (16.4)	297 (17.7)	144 (7.3)	143 (7.3)	432 (100.0)	432 (100.0)	0		0
50-74	1315 (78.6)	1307 (78.1)	1670 (84.8)	1669 (84.8)	0	0	2654 (93.1)		2651 (93.0)
≥75	85 (5.1)	70 (4.2)	155 (7.9)	157 (8.0)	0	0	197 (6.9)		200 (7.0)
Charlson-Deyo Comorbidity Score									
0	1405 (83.9)	1369 (81.8)	1670 (84.8)	1672 (84.9)	394 (91.2)	382 (88.4)	2424 (85.0)		2458 (86.2)
1	224 (13.4)	259 (15.5)	258 (13.1)	246 (12.5)	33 (7.6)	41 (9.5)	380 (13.3)		345 (12.1)
2	45 (2.7)	46 (2.7)	41 (2.1)	51 (2.6)	5 (1.2)	9 (2.1)	47 (1.6)		48 (1.7)
Histological profile									
Ductal or lobular	1597 (95.4)	1601 (95.61	1900 (96.5)	1907 (96.9)	407 (94.2)	403 (93.3)	2767 (97.1)		2769 (97.1)
Others	77 (4.6)	73 (4.41	69 (3.5)	62 (3.1)	25 (5.8)	29 (6.7)	84 (2.9)		82 (2.9)
Grade									
Well differentiated	40 (2.4)	36 (2.2)	68 (3.5)	70 (3.6)	16 (3.7)	16 (3.7)	85 (3.0)		84 (2.9)
Moderately differentiated	438 (26.2)	426 (25.4)	586 (29.8)	589 (29.9)	106 (24.5)	123 (28.5)	839 (29.4)		837 (29.4)
Poorly differentiated	1083 (64.7)	1086 (64.9)	1253 (63.6)	1246 (63.3)	282 (65.3)	248 (57.4)	1829 (64.2)		1834 (64.3)
Other	3 (0.2)	3 (0.2)	1 (0.1)	3 (0.2)	1 (0.2)	5 (1.2)	1 (0.0)		1 (0.0)
Not available	110 (6.6)	123 (7.3)	61 (3.1)	61 (3.1)	27 (6.3)	40 (9.3)	97 (3.4)		95 (3.3)
Race									
White	1289 (77.0)	1271 (75.9)	1641 (83.3)	1650 (83.8)	330 (76.4)	313 (72.5)	2386 (83.7)		2351 (82.5)
Black	298 (17.8)	296 (17.7)	293 (14.9)	290 (14.7)	77 (17.8)	92 (21.3)	402 (14.1)		404 (14.2)
Others	74 (4.4)	89 (5.3)	32 (1.6)	25 (1.3)	22 (5.1)	25 (5.8)	58 (2.0)		91 (3.2)
Not available	13 (0.8)	18 (1.1)	3 (0.2)	4 (0.2)	3 (0.7)	2 (0.5)	5 (0.2)		5 (0.2)
Year									
2010-2012	733 (43.8)	734 (43.8)	989 (50.2)	979 (49.7)	223 (51.6)	242 (56.0)	1323 (46.4)		1310 (45.9)
2013-2015	941 (56.2)	940 (56.2)	980 (49.8	990 (50.3)	209 (48.4)	190 (44.0)	1528 (53.6)		1541 (54.1)
pT staging									
T1a	1674 (100.0)	1674 (100.0)	0	0	242 (56.0)	229 (53.0)	1026 (36.0)		1032 (36.2)
T1b	0	0	1969 (100.0)	1969 (100.0)	190 (44.0)	203 (47.0)	1825 (64.0)		1819 (63.8)
Lymph nodes examined, No.									
0-2	801 (47.8)	814 (48.6)	1112 (56.5)	1094 (55.6)	215 (49.8)	204 (47.2)	1550 (54.4)		1578 (55.3)
>2	852 (50.9)	824 (49.2	851 (43.2)	870 (44.2)	213 (49.3)	220 (50.9)	1294 (45.4)		1267 (44.4)
Not available	21 (1.3)	36 (2.2)	6 (0.3)	5 (0.3)	4 (0.9)	8 (1.9)	7 (0.2)		6 (0.2)
Surgery									
Lumpectomy	1037 (61.9)	1032 (61.6)	1364 (69.3)	1359 (69.0)	184 (42.6)	197 (45.6)	1984 (69.6)		1967 (69.0)
Mastectomy	637 (38.1)	642 (38.4)	605 (30.7)	610 (31.0)	248 (57.4)	235 (54.4)	866 (30.4)		883 (31.0)
Others	0	0	0	0	0	0	1 (0.0)		1 (0.0)
Margin									
Negative	1633 (97.6)	1618 (96.7)	1947 (98.9	1950 (99.0)	418 (96.8)	404 (93.5)	2827 (99.2)		2828 (99.2)
Positive	31 (1.9)	40 (2.4)	19 (1.0)	18 (0.9)	11 (2.5)	19 (4.4)	19 (0.7)		20 (0.7)
Not available	10 (0.6)	16 (1.0	3 (0.2)	1 (0.1)	3 (0.7)	9 (2.1)	5 (0.2)		3 (0.1)
Radiation									
None	647 (38.6)	643 (38.4)	719 (36.5)	720 (36.6)	260 (60.2)	251 (58.1)	983 (34.5)		998 (35.0)
External beam	973 (58.1)	976 (58.3)	1167 (59.3)	1166 (59.2)	163 (37.7)	167 (38.7)	1752 (61.5)		1747 (61.3)
Others	51 (3.0)	52 (3.1)	83 (4.2)	82 (4.2)	8 (1.9)	10 (2.3)	115 (4.0)		106 (3.7)
Not available	3 (0.2)	3 (0.2)	0	1 (0.1)	1 (0.2)	4 (0.9)	1 (0.0)		0
Radiation dose, median (IQR), Gy	60.4 (52.6-61.2)	60.4 (57.0-61.2)	60.4 (52.6-62.0)	60.4 (59.4-62.4)	60.4 (59.0-61.2)	60.4 (60.0-63.0)	60.4 (52.6-62.0)		60.4 (57.0-62.0)
Readmission within 30 d									
None	1597 (95.4)	1617 (96.6)	1929 (98.0)	1927 (97.9)	417 (96.5)	401 (92.8)	2792 (97.9)		2787 (97.8)
Unplanned	15 (0.9)	16 (1.0)	12 (0.6)	13 (0.7	2 (0.5)	6 (1.4)	14 (0.5)		14 (0.5)
Planned	40 (2.4)	26 (1.6)	18 (0.9)	17 (0.9)	6 (1.4)	12 (2.8)	34 (1.2)		38 (1.3)
Others	2 (0.1)	1 (0.1)	1 (0.1)	1 (0.1)	7 (1.6)	13 (3.0)	11 (0.4)		12 (0.4)
Not available	20 (1.2)	14 (0.8)	9 (0.5)	11 (0.6)	0	0	0		0
Postoperative inpatient duration, median (IQR), d	0 (0-1)	0 (0-1)	0 (0-1)	0 (0-1)	1 (0-2)	1 (0-2)	0 (0-1)		0 (0-1)

Abbreviation: IQR, interquartile range.

^a^ Matching was performed for characteristics including treatment facility volume, facility type, age, race, comorbidity score, year of diagnosis, histological profile, tumor grade, number of lymph nodes examined, type of surgery and radiation, surgical margin, radiation dose, postoperative readmissions, and duration of postoperative inpatient admission.

**Figure.**
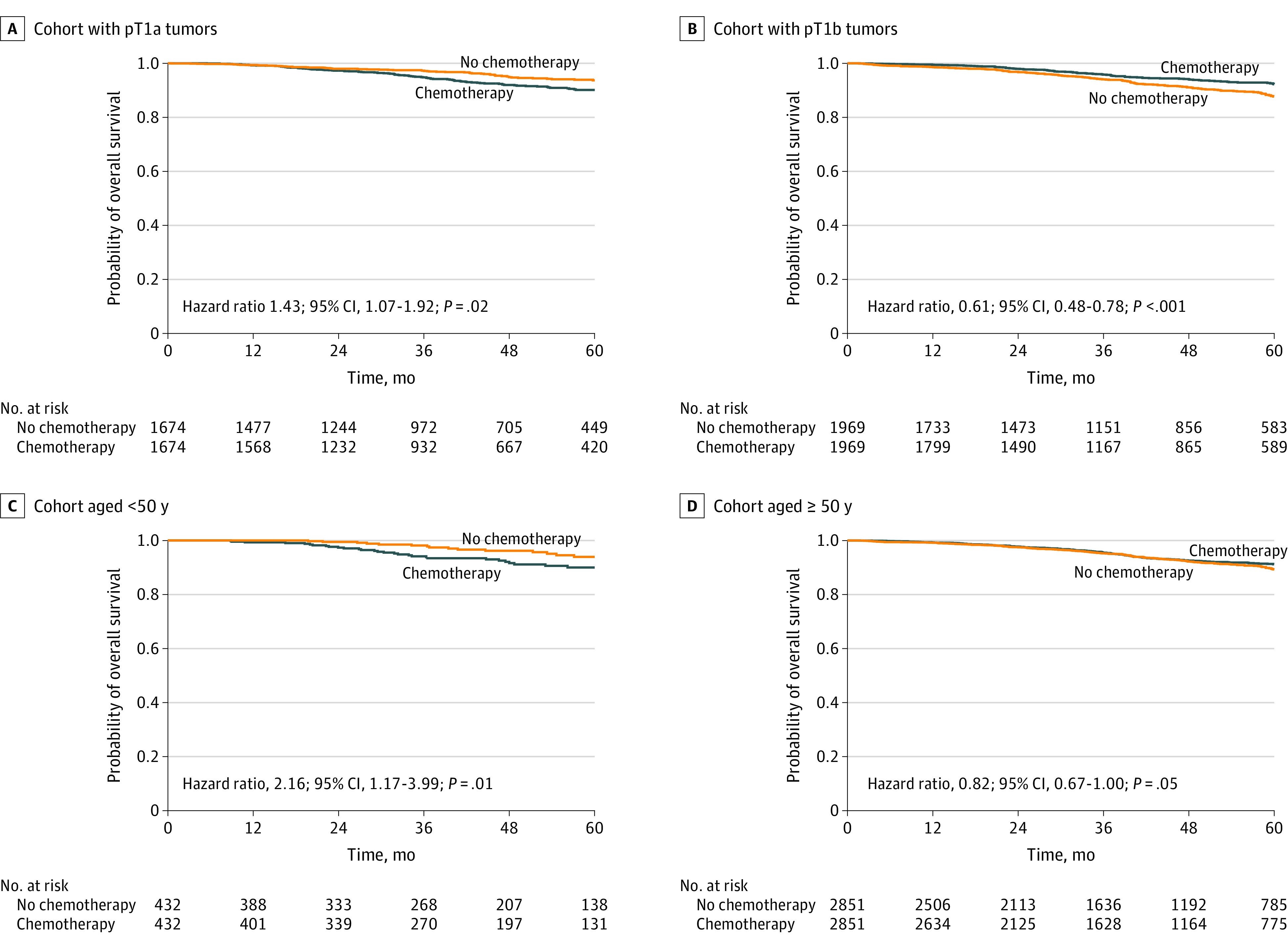
Kaplan-Meier Survival Curves After Matching Panels A and B include patients with pT1a tumors (A) and pT1b tumors (B) who received or did not receive chemotherapy. Panels C and D include patients younger than 50 years (C) and 50 years or older (D) who received or did not receive chemotherapy.

## Discussion

To our knowledge, this is the largest observational cohort study of small TNBC evaluating the role of chemotherapy. In our study, chemotherapy was associated with significant survival benefits in patients aged 50 years or older who had pT1b tumors. These pT1b tumors have 5-year distant recurrence of up to 10%,^2^ perhaps explaining the survival benefits with chemotherapy in this subgroup. Surprisingly, chemotherapy was associated with worse mortality for patients younger than 50 years who had pT1a tumors. Reasons for this observation are unclear, although it may be in part because of worse chemotherapy toxic effects outweighing its benefits for pT1a tumors^4^ and younger cohorts receiving chemotherapy with more aggressive tumor biology.^5^ Pertinent variables, such as performance status, tumor recurrence, and toxicity, were unavailable in the National Cancer Database, resulting in unmeasured confounding despite matching. Nevertheless, as there are favorable outcomes regardless of chemotherapy use and given the risk of worsened mortality, the avoidance of chemotherapy may be considered for those younger than 50 years and with pT1a TNBC.

## Notes

Supplement. **eAppendix.** Supplemental Methods
