# Supplementary material for: Association of Adjuvant Chemotherapy With Overall Survival Among Women With Small, Node-Negative, Triple-Negative Breast Cancer
Source: JAMA Netw Open. 2020 Sep 14;3(9):e2016247. doi: 10.1001/jamanetworkopen.2020.16247 (PMC7490645; doi:10.1001/jamanetworkopen.2020.16247)
Supplement: Supplement. — eAppendix. Supplemental Methods [file jamanetwopen-e2016247-s001.pdf]

Supplementary Online Content

Oladeru OT, Singh AK, Ma SJ. Association of adjuvant chemotherapy with overall survival among women with small, node-negative, triple-negative breast cancer. *JAMA Netw Open*. 2020;3(9):e2016247. doi:10.1001/jamanetworkopen.2020.16247

eAppendix. Supplemental Methods

This supplementary material has been provided by the authors to give readers additional information about their work.

© 2020 Oladeru OT et al. *JAMA Network Open.*

Supplemental Methods

Methods

Approval for our study was obtained from our institutional review board (BDR-122819) and the analysis was performed from March to May 2020. In addition, this study follows the Strengthening the Reporting of Observational Studies in Epidemiology (STROBE) reporting guideline.

The follow up period for our study cohort was until the end of 2017. The primary endpoint was overall survival (OS), defined as the time interval between diagnosis and the last follow-up or death. Categorical and continuous variables were compared using Fisher exact test and Mann-Whitney U test, respectively. Cox proportional hazard multivariable analysis (MVA) model was constructed including all statistically significant variables from the Cox univariable analysis followed by a backward stepwise elimination. Variables in the final model include facility type, facility volume, age, race, Charlson-Deyo comorbidity score (CDS), year of diagnosis, and type of radiation received. Assumptions of Cox proportional hazard model were verified graphically based on Schoenfeld residual method. The potential interaction of chemotherapy with tumor size and age were examined by adding interaction terms to Cox proportional hazard MVA final model. The final model was re-assessed for tumor size and age subgroups to compare the magnitude of the effect of chemotherapy.

In addition, to address the selection bias, propensity score matching was performed using variables from the Cox proportional hazard MVA final model and other clinically relevant variables. Matched variables include facility volume, facility type, age, CDS, histology, race, year of diagnosis, tumor size, number of lymph nodes examined, surgery, surgical margin, radiation, radiation dose, inpatient readmission within 30 days postoperatively, and duration of postoperative inpatient admission. The matched cohorts from tumor size and age subgroups were examined for survival outcomes.

All p values were two-sided and p values less than 0.05 were considered statistically significant.

© 2020 Oladeru OT et al. *JAMA Network Open.*
